# Comment on ‘COVID‐19: a major cause of cachexia and sarcopenia’ by Morley *et al*.

**DOI:** 10.1002/jcsm.12648

**Published:** 2020-12-03

**Authors:** Ling Zhou, Cunming Liu, Chun Yang

**Affiliations:** ^1^ Department of Anesthesiology and Perioperative Medicine The First Affiliated Hospital of Nanjing Medical University Nanjing Jiangsu Province China

We read with great interest the letter by Morley *et al*., in which the authors concluded that coronavirus disease 2019 (COVID‐19) might be a major cause of cachexia and sarcopenia.^1^ Although the paper possessed convincing evidences, several issues still need to be addressed.

Firstly, Morley *et al*. mentioned that the angiotensin‐converting enzyme 2 (ACE2), a receptor for severe acute respiratory syndrome coronavirus 2 (SARS‐CoV‐2), expressed in skeletal muscle, which might partially explain the symptoms of myalgia and muscle wasting in COVID‐19. However, according to the current results, although ACE2 is widely distributed in the human body, the protein level of ACE2 has not been detected in skeletal muscle, and the RNA level of ACE2 is also extremely low (0.7 normalized expression).^2^ We therefore think that myalgia and muscle wasting in COVID‐19 patients may have little relationship with the direct invasion of SARS‐CoV‐2 via ACE2 in skeletal muscle. Meanwhile, mammalian target of rapamycin complex 1 (mTORc1) is an important regulator of muscle protein synthesis (MPS) and muscle protein turnover. Although mTORc1 is hyperactivated in elderly individuals with sarcopenia, mTORc1 resistance occurs during this period.^3^, ^4^ Given that most patients with severe symptoms are elderly individuals, mTORc1 resistance may exacerbate the decrease in muscle protein synthesis.

Secondly, the authors mentioned that COVID‐19 patients suffer from anorexia, weight loss, and low albumin, known as symptoms of cachexia and sarcopenia. We also recognized that muscle wasting was common in severe COVID‐19 cases. However, COVID‐19 for cachexia and sarcopenia: culprit, accomplice, or bystander? We think COVID‐19 as a trigger, rather than a main cause. Isolation measures, such as travel bans and enforced quarantine, have the potential to cause reduced physical activity, increased appetite, and overconsumption of food, which exacerbates muscle loss and increases fat mass (sarcopenic obesity).^4^ Studies have shown that a short‐term immobilization (2 days) can cause a loss of muscle volume by 1.7%, and it suffers a greater loss (5.5% of muscle volume) after 7 days.^5^ Moreover, skeletal muscle secretes a variety of myokines, including interleukin (IL)‐6 and IL‐7,^6^ which would greatly affect the immunity. The cytokine storm observed in severe COVID‐19 patients would damage tissues directly, as well as inhibit MPS by disturbing hormones regulating muscle growth, such as testosterone and insulin‐like growth factor 1.

COVID‐19 patients with cachexia and sarcopenia are mostly in the severe scenario. Fortunately, age‐related sarcopenia is due to the reduction of type II muscle fibres, which can be improved with regular resistance exercise^7^ and higher protein intake.^8^ In addition, direct sunlight is beneficial for the synthesis of vitamin D to enhance protein synthesis.

In conclusion, cachexia and sarcopenia in patients with COVID‐19 should arise widespread attentions. More detailed and well‐designed studies are needed in exploring the association between COVID‐19 and cachexia and sarcopenia. Meanwhile, effective prevention and rehabilitation strategies should urgently be taken to protect the susceptible individuals.
